# Pediatric neurosurgical-oncology scope and management paradigms in Sub-Saharan Africa: a collaboration among 7 referral hospitals on the subcontinent

**DOI:** 10.3389/fonc.2023.1257099

**Published:** 2023-11-01

**Authors:** Joseline Haizel-Cobbina, Silky Chotai, Jason Labuschagne, Addisalem Belete, Yordanos Ashagere, Hamisi K. Shabani, William Copeland, Kachinga Sichizya, Misbahu Haruna Ahmad, Frank Nketiah-Boakye, Michael C. Dewan

**Affiliations:** ^1^ Department of Neurosurgery, Vanderbilt University Medical Center, Nashville, TN, United States; ^2^ Vanderbilt Institute for Global Health, Vanderbilt University Medical Center, Nashville, TN, United States; ^3^ Department of Surgery, Cape Coast Teaching Hospital, Cape Coast, Ghana; ^4^ Department of Paediatric Neurosurgery, Nelson Mandela Children’s Hospital, Johannesburg, South Africa; ^5^ Department of Neurosurgery, Zewditu Memorial Hospital, Addis Ababa, Ethiopia; ^6^ Department of Neurosurgery, Muhimbili Orthopaedic Institute, Dar es Salaam, Tanzania; ^7^ Department of Neurosurgery, Tenwek Mission Hospital, Bomet, Kenya; ^8^ Department of Neurosurgery, University Teaching Hospital, Lusaka, Zambia; ^9^ Department of Neurosurgery, Aminu Kano Teaching Hospital, Kano, Nigeria; ^10^ Department of Neurosurgery, Komfo Anokye Teaching Hospital, Kumasi, Ghana

**Keywords:** pediatric, neurosurgical-oncology, sub-Saharan Africa, CNS tumor, surgery wait time, postoperative length of hospital stay

## Abstract

**Background:**

Understanding of the epidemiology and biology of pediatric CNS tumors has advanced dramatically over the last decade; however there remains a discrepancy in the understanding of epidemiologic data and clinical capacity between high- and lower-income countries.

**Objective:**

We collected and analyzed hospital-level burden and capacity-oriented data from pediatric neurosurgical oncology units at 7 referral hospitals in Sub-Saharan Africa (SSA).

**Methods:**

A cross sectional epidemiological survey was conducted using REDCap at the 7 SSA sites, capturing 3-month aggregate data for patients managed over a total of 9 months. Descriptive statistical analyses for the aggregate data were performed.

**Results:**

Across the neurosurgical spectrum, 15% of neurosurgery outpatient and 16% of neurosurgery operative volume was represented by pediatric neuro-oncology across the 7 study sites. Eighty-six percent and 87% of patients who received surgery underwent preoperative CT scan and/or MRI respectively. Among 312 patients evaluated with a CNS tumor, 211 (68%) underwent surgery. Mean surgery wait time was 26.6 ± 36.3 days after initial presentation at the clinic. The most common tumor location was posterior fossa (n=94, 30%), followed by sellar/suprasellar region (n=56, 18%). Histopathologic analysis was performed for 189 patients (89%). The most common pathologic diagnosis was low grade glioma (n=43, 23%), followed by medulloblastoma (n=37, 20%), and craniopharyngioma (n=31, 17%). Among patients for whom adjuvant therapy was indicated, only 26% received chemotherapy and 15% received radiotherapy.

**Conclusion:**

The histopathologic variety of pediatric brain and spinal tumors managed across 7 SSA referral hospitals was similar to published accounts from other parts of the world. About two-thirds of patients received a tumor-directed surgery with significant inter-institutional variability. Less than a third of patients received adjuvant therapy when indicated. Multi-dimensional capacity building efforts in neuro-oncology are necessary to approach parity in the management of children with brain and spinal tumors in SSA.

## Introduction

Central nervous system (CNS) tumors pose a significant public health burden worldwide and is reported to affect 7-11 persons per 100,000 person-years (1, 2). The 2016 global burden of disease collaborative report estimated 330,000 new cases of CNS cancer each year. Moreover, the age-standardized incidence rate of CNS cancers has increased by 17.3% from 1990 to 2016 (2). Pediatric brain and spine tumors are a subset of CNS cancers, representing roughly 17% of all childhood malignancies (3). They are the most common solid tumors of childhood and are the leading cause of cancer-related death in children and adolescents (4–6).

Epidemiological data on burden of pediatric brain and spine tumors has become increasingly available in recent years with a higher incidence reported in high-income countries (HIC) compared to low- and middle-income countries (LMICs) (4, 6–18). This discrepancy can be attributed to poor access to care, lack of imaging resources, inadequate histological diagnostic capabilities, and unreliable or nonexistent tumor registries – all resulting in underreporting in LMIC (11, 17, 19). Over the last two decades substantial resources have been mobilized in HIC for diagnosis, management and research to better understand these tumors (13, 20–22). Advances in managing these tumors are such that most children diagnosed with a CNS malignancy in a well-resourced healthcare system have an option to be enrolled in one or more trials offering cutting edge therapies. This is not the case for children in LMICs where cancer-related mortality is much greater (9, 19).

Before we can begin to invest in the infrastructure to support translational medicine and trial networks in LMICs, understanding the neuro-oncology burden and existing resources is fundamental. To better understand the hospital-level burden of disease and capacity, data was collected across 7 national referral hospitals in Sub-Saharan Africa (SSA) via a cross-sectional survey. In this study, we set out to report the burden of pediatric CNS tumors, their histologic subtype, and the basic resource availability to manage these patients.

## Methods

A participatory research approach was employed in our research driven by the need to engage relevant stakeholders involved in pediatric neuro-oncology care in SSA. Potential research collaborators in SSA were identified through professional contacts in the academic neuroscience space. An invitation was extended to the identified neurosurgeons in East Africa, West Africa, and Southern Africa to participate in this study. A study team was assembled based on responses received which included 7 neurosurgeons from 7 referral hospitals in SSA, their respective neurosurgery teams and clinical care coordinators at the study site who assisted with data collection. Three neurosurgeons invited could not participate in the study due challenges related to participatory bandwidth or institutional IRB processes.

A 43-item survey was designed collectively by the study team to assess the hospital-level burden and capacity data from pediatric neurosurgical oncology units at the 7 referral hospitals in SSA (**Supplementary File 1**).

**Figure 1:**
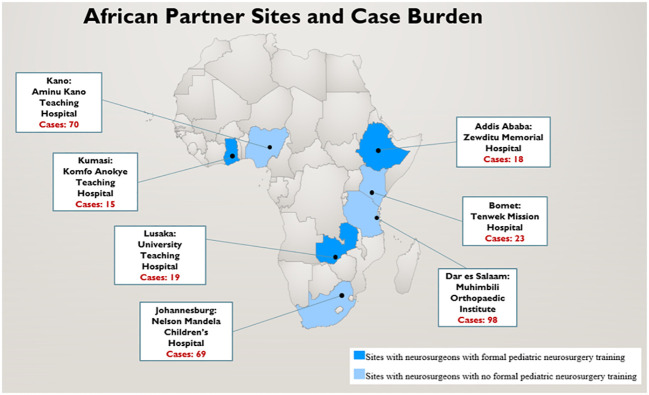
Map showing location of study sites in Sub-Saharan Africa and case burden.

Institutional review board (IRB) approval was obtained from all 7 SSA sites, as well as the North American coordinating site, Vanderbilt University Medical Center. The seven SSA study sites included: Aminu Kano Teaching Hospital (AKTH), Kano, Nigeria; Komfo Anokye Teaching Hospital (KATH), Kumasi, Ghana; Nelson Mandela Children’s Hospital (NMCH), Johannesburg, South Africa; Tenwek Mission Hospital(TMH), Bomet, Kenya; Zewditu Memorial Hospital (ZMH), Addis Ababa, Ethiopia; University Teaching Hospital (UTH), Lusaka, Zambia; and Muhimbili Orthopedic Institute (MOI), Dar es Salaam, Tanzania (**Figure 1**). Responses to all the survey questions was obtained by retrospective review of aggregate hospital data during the study period (January 2021 – October 2021). Therefore, identity of patients cannot be ascertained based on information collected by investigators.

The Research Electronic Data Capture (REDCap) tool was used for the cross-sectional epidemiological survey data collection (23). Over the 9-month study period, the REDCap survey was completed by the participating neurosurgeon at each of the 7 sites at three-month intervals to capture aggregate retrospective data of all pediatric CNS tumor cases diagnosed and/or managed over the preceding 3 months. Participating neurosurgeons provided information related to their surgical training, primary hospital and neurosurgical practice. Neuro-oncologic information included that related to tumor location and histopathology, operative details, postoperative management, adjuvant therapy, and follow-up practices. Data was obtained from outpatient and inpatient records, surgical case logs, oncology case logs, and hospital administrative data. The REDCap survey was completed at 3-month intervals for ease of data collection. The complete survey tool is included in the appendix (**Supplementary File**).

## Statistical analysis

Descriptive statistics were pooled across the 7 SSA sites. Continuous data were presented as mean, standard deviation, median and range. Categorical data were presented as frequency and percentage. All statistical analyses were performed using the SPSS version 27 (IBM, Chicago, Inc).

## Results

### Partner site details and resource availability

Responses were obtained from neurosurgeons at all 7 partnering sites. Each neurosurgeon reported 3-monthly retrospective aggregate pediatric CNS tumor patient data and diagnostic and management capacity at their respective hospitals over the 9-month study period. The three neurosurgeons from UTH, ZMH, and KATH received formal pediatric neurosurgery training. Each site reported a median of 2 neurosurgeons (Range, 1-4) with pediatric neurosurgery practice. During the 9-month window of data collection, a total of 312 patients with pediatric brain or spinal tumors were diagnosed and evaluated in the clinic, emergency department, or ward at the 7 partner sites (**Figure 1**).

**Figure 2:**
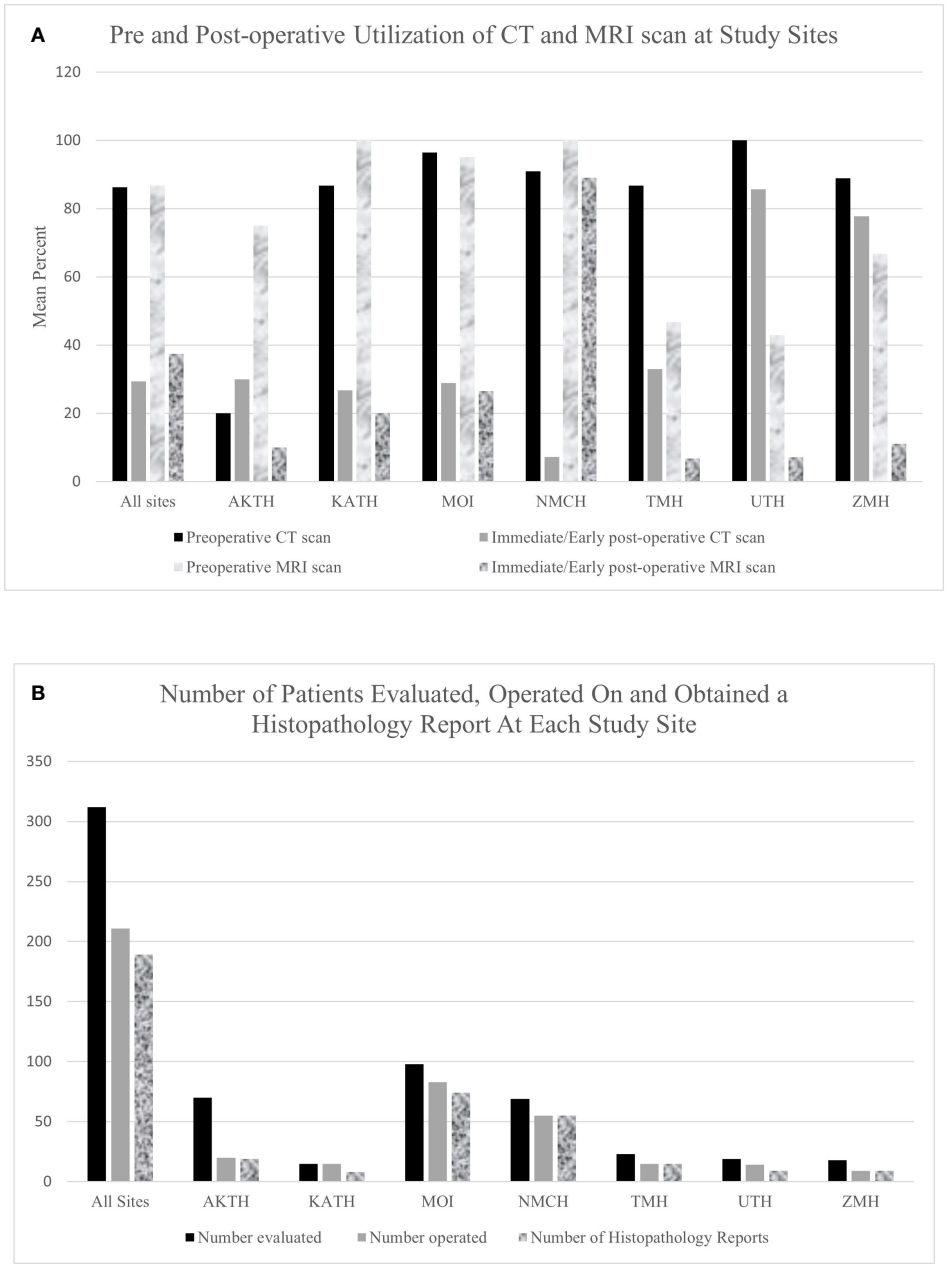
**(A)** Bar graph showing pre and post operative utilization of CT and MRI scan at study sites. **(B)** A Bar Graph showing the number of patients evaluated and operated on and obtained a histopathology report at all study sites.

Cumulative across 7 sites, 14.8% of neurosurgery outpatient and 15.8% of neurosurgery operative volume was represented by pediatric neuro-oncology at the partner sites. Computed tomography and/or magnetic resonance imaging was available at all but one site which had no diagnostic imaging modality available. Six sites had a radiologist on site to interpret imaging findings but none of the six had a staff neuroradiologist. One site (TMH) had no radiologist on site even though there is a CT and MRI scanner available, and depended on an international team of radiologists from the United States (US) who provided teleradiology consults. Among patients who underwent a surgical intervention, 86% and 87% of patients underwent preoperative CT and MRI, respectively. Post-operatively, 29% and 37% of patients underwent CT and MRI, respectively, in the immediate/early post-operative period (**Figure 2A**).

### Surgical management

Of the 312 patients evaluated, 211 (67.6%) patients underwent tumor-directed surgery and 16 (5.1%) were referred to other centers (**Figure 2B**). There was wide variability in the proportion of patients operated on at each hospital. At KATH, 100% of patients evaluated underwent surgery, whereas only 28% did so at AKTH. Among the 211 patients who underwent surgery, 178 patients (84.4%) underwent resection of tumor, 23 (10.9%) underwent biopsy and for 10 (4.7%) the type of tumor-directed surgery was unspecified. Among all presenting tumors, the most common tumor location was posterior fossa (n=94, 30.1%), followed by sellar/suprasellar (n=56, 17.9%), ventricular (n=29, 9.3%), brainstem (n=22, 7.1%), and spinal (n=22, 7.1%).

**Figure 3:**
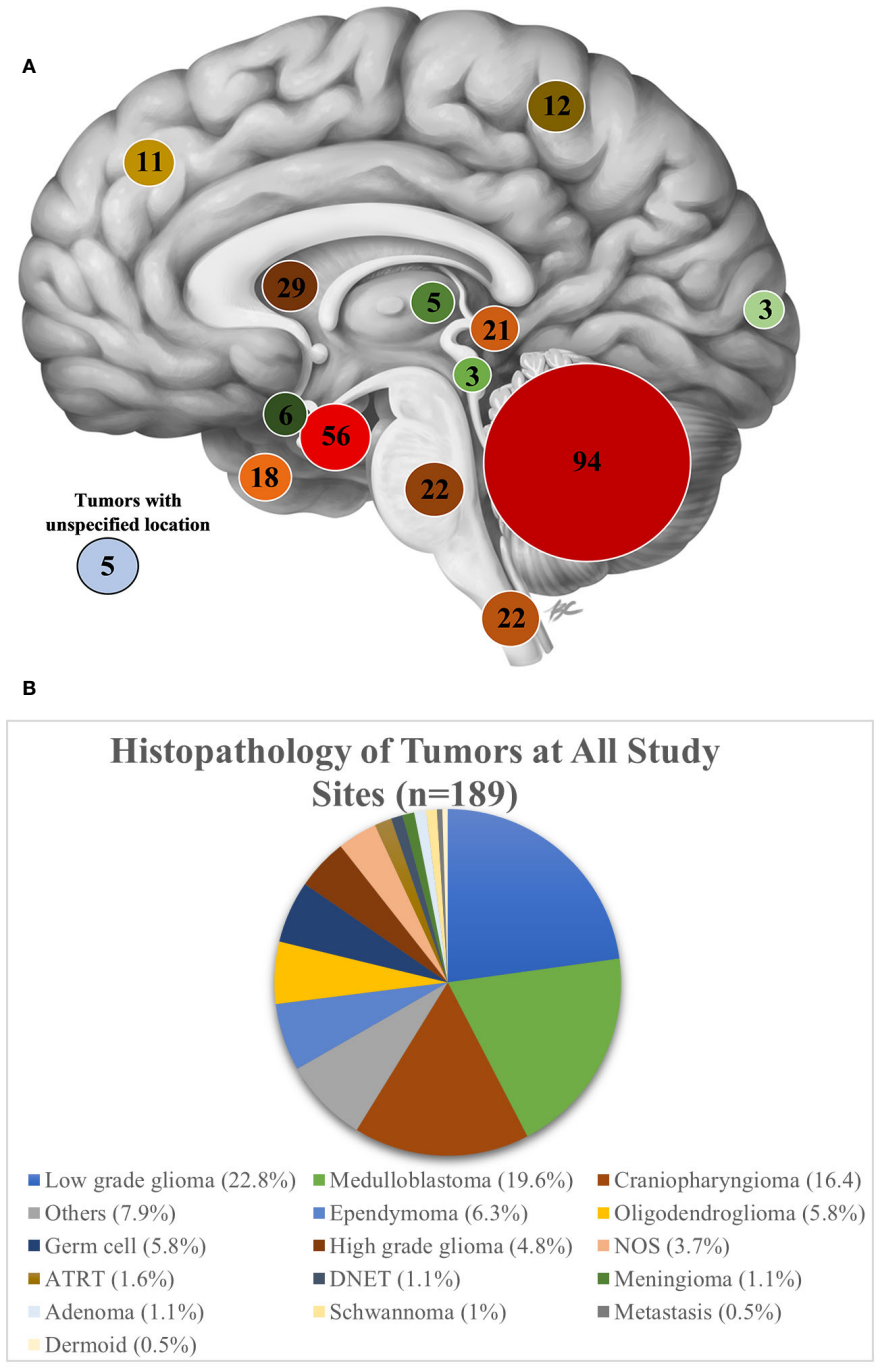
**(A)** A sagittal view of the brain showing the number of patients evaluated on based on tumor location at all study sites. *Image Credit: B Cheung Biomedical Illustration, 2020* (23) **(B)** Histopathology of tumors at all study sites.

The most common tumor location for patients who underwent surgery was posterior fossa (n=72, 34.1%), followed by sellar/suprasellar (n=38, 18.0%), spinal (n=20, 9.4%) and ventricular (n=19, 9.0%) (**Figure 3A**).

Among 211 patients who underwent tumor-directed surgery, 85 (40.3%) had a CSF diversion either via shunt or endoscopic third ventriculostomy (ETV). Of the 101 patients who did not have tumor-directed surgery, 68 underwent a CSF diversion alone.

### Tumor histopathology and adjuvant care

None of the seven partner sites had neuropathology expertise to interpret the histopathology, instead all tissue samples and histopathology slides were reviewed by a general pathologist. Ten percent of cases did not receive a histopathology report. Of the 90% of cases which received a report, a histopathological diagnosis was made for 88.4%, while for the remaining specimens, the diagnosis was either unspecified neoplasm (NOS) (3.7%) or classified as “other” (7.9%). As an aggregate across all sites, the most common pathologic diagnosis was low grade glioma (22.9%), followed by medulloblastoma (19.7%), and craniopharyngioma (16.5%) (**Figure 3B**). Among individual sites, however, the proportion of tumor histologies varied. Low grade glioma was the most frequently reported tumor at 4 of the 7 sites (KATH, 62.5%; MOI, 31%; ZMH, 22%; NMCH, 18.2%). Craniopharyngioma was the most common tumor subtype managed at AKTH (31.6%). At NMCH, medulloblastoma (14.5%) was equally as common as craniopharyngioma (14.5%). The sites UTH, ZMH, and TMH noted somewhat equal distribution of tumor pathology.

Among patients for whom adjuvant therapy was indicated, only 26% received adjuvant chemotherapy and 15% received adjuvant radiotherapy. There was variability among partnering sites in aggregate percent of patients who received adjuvant chemotherapy and radiotherapy. Most sites relied on a pediatric oncologists or medical oncologists to provide adjuvant therapy to pediatric neurosurgical-oncology patients.

### Surgery wait times and length of hospital stay

The mean wait time to surgery for patients who initially presented to the clinic was 26.6 ± 36.3 days and median wait time was 17 days (Range, 5-180). For patients admitted to the emergency or ward on initial presentation, the mean wait time to surgery was 18.2 ± 37.9 days and median wait time was 9 days (Range, 3-180). For patients undergoing surgery, the mean preoperative length of hospital stay (LOS) was 10.6 ± 8.4 days and median preoperative LOS was 9 days (Range, 2-30) and the mean post-operative length of hospital stay (LOS) was 10.4 ± 6.0 days and median post-operative LOS was 8 days (Range, 4-28). The mean post-operative ICU stay (LOS) was 3.4 ± 2.4 days and median post-operative ICU stay was 3 days (Range, 0-12).

**Figure 4:**
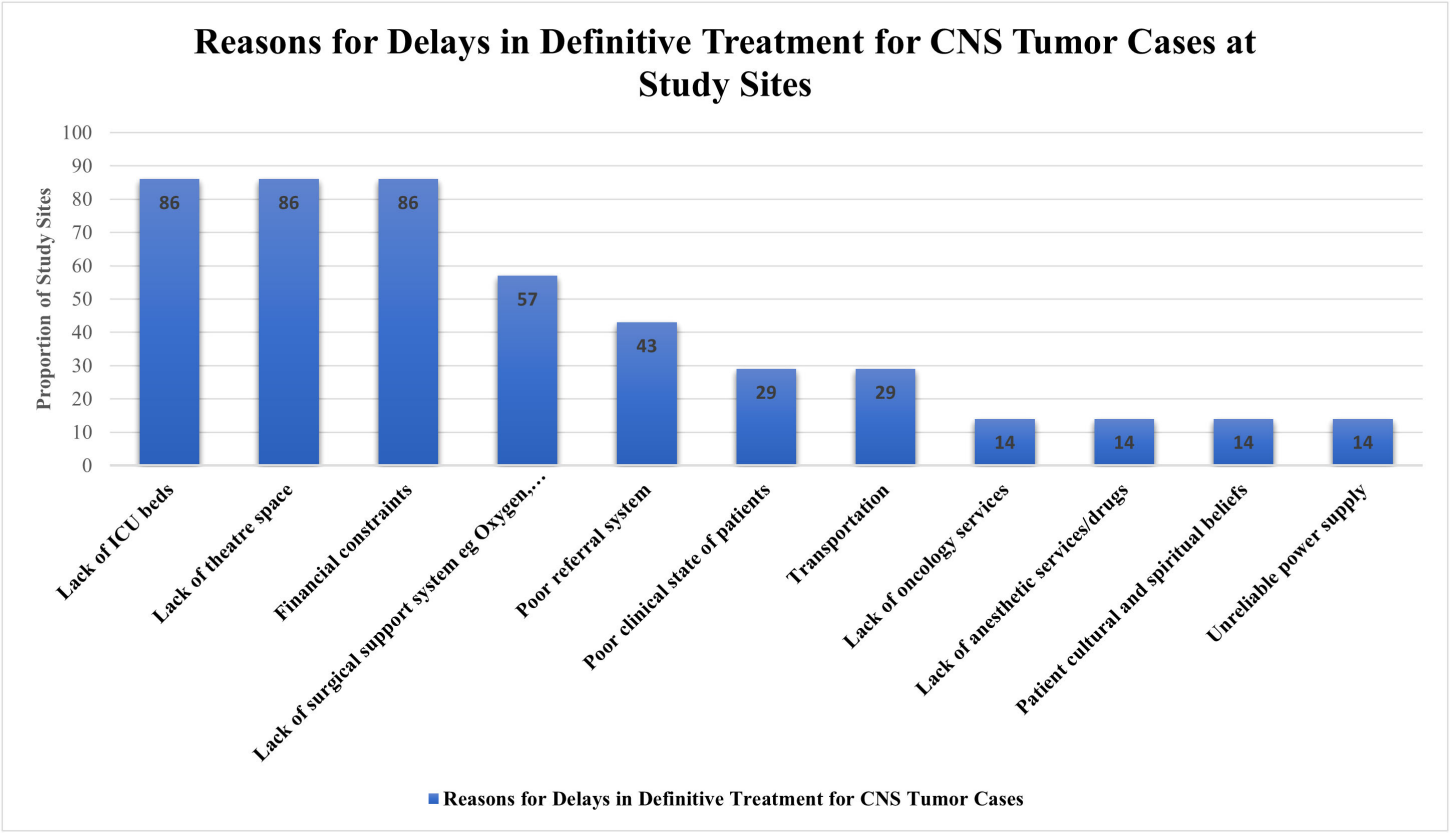
A Bar Graph showing the reasons for delay in definitive treatment for CNS tumor cases at study sites.

A mean 26.4% (SD: 29.4%) patients who were candidates for tumor surgery did not undergo surgery due to inadequate resources. This was variable among the partner sites; TMH reported 0% of such patients and AKTH reported 73.7% patients who did not undergo surgery due to inadequate resources. Apart from KATH, all other sites reported having a surgery waiting list. Some of the reasons given for either delays in definitive treatment for CNS tumor cases or patients not receiving surgery reported by sites include lack of ICU beds (86%), lack of operating room space (86%), financial constraints (86%), lack of support system e.g., imaging modalities, blood and blood products (57%) (**Figure 4**). Twenty-four patients on the waiting list were reported to have died before they could have surgery. AKTH reported the highest number of deaths (8), followed by NMCH (7), MOI (5), ZMH (3), and UTH (1).

### Surgical complications, Follow-up, and COVID-19 impact

Twenty-eight (13%) patients who had surgery experienced minor complications such as surgical site infection, wound dehiscence, and minor/transient neurological deficit. Major complications, including hemiparesis, permanent aphasia, or post-op meningitis were observed in 14 (7%). Across the 7 centers and among the 211 surgical patients, the surgical mortality was 2.4%.

Mean 73% (SD:32.1%) of patients received early postoperative follow-up (2-6 weeks post hospital discharge) and 51.7% (SD:34.9%) received mid-term follow-up (>3 months post hospital discharge).

The COVID-19 pandemic had a variable impact on neurosurgical-oncology volume across the 7 sites. UTH reported a 25%-50% reduction, NMCH reported a 25-75% reduction, AKTH reported a 50% reduction, and ZMH reported >75% reduction over the 9-month study period. Three sites (TMH, KATH, and MOI) reported no significant reduction in CNS tumor case volume due to the COVID-19 pandemic. AKTH reported doctors’ industrial action (labor strike) as an additional reason for reduction in CNS tumor case volume during which there was cancellation of clinic consultations and non-emergent surgeries. Five treatment centers, KATH, MOI, TMH, UTH, AKTH had either a hospital-based or population-based tumor registry.

## Discussion

An understanding of the basic epidemiological, clinical, and capacity data is necessary to guide the expansion of pediatric neuro-oncologic services in SSA, where hospital-level data is largely unknown. In this study, we aggregated cross-sectional data from 7 national referral sites in SSA to gain an understanding of the CNS tumor volume, location, and pathology, as well as neurosurgical resource availability and utilization for children with brain and spinal tumors.

Pediatric neuro-oncology forms a considerable proportion of the outpatient and operative volume at the seven national referral sites surveyed. At most sites, these cases are managed by general neurosurgeons due to the lack of fellowship trained pediatric neurosurgeons and neurosurgical oncologists. Despite the increasing burden of cases, pediatric neuro-oncology care is less prioritized in Africa as most treatment centers remain under-resourced.

Pediatric neuro-oncology care is complex and often require multidisciplinary approach. Based on the location radiographic characteristics of the tumor and presenting symptoms, surgical excision or biopsy is indicated for most pediatric brain and spine tumors. In our study, while a majority of the patients underwent preoperative CT or MRI imaging under the auspices of the treating neuro-oncologic team, 10-20% of patients had to arrange imaging elsewhere at a non-affiliated site, likely delaying care. Neuronavigation, intra-operative imaging modalities, and cortical mapping technology are scarce leaving most neurosurgeons in SSA to rely on anatomical landmarks alone to perform tumor surgeries (24). Less than half of patients underwent immediate/early post-operative CT or MRI imaging. Without an objective understanding of extent of resection, advising adjuvant therapy and disease monitoring via surveillance imaging becomes both challenging and speculative (25).

Analogous to prior reports from studies across the globe, a wide spectrum of pediatric brain and spine tumors were managed at the seven national referral sites in SSA (2, 9, 10, 17, 20, 21, 26, 27). Low-grade glioma was the most common subtype followed by craniopharyngioma and medulloblastoma. Similarly, Stagno et al. in a retrospective series of 172 Ugandan patients operated on over a 10-year period, found the most common tumor to be low grade glioma, followed by ependymoma, craniopharyngioma, choroid plexus papilloma and medulloblastoma (17). However, no cases of choroid plexus papilloma were noted in our study. In a multicenter retrospective study from Morocco, medulloblastoma was the most common histopathological subtype followed by low grade glioma, ependymoma and craniopharyngioma (14). The variation in pathological subtypes of tumor might be attributed to the different regional biology, different presentation patterns, or simply a limited dataset. Among the seven referral sites in our study, similar variabilities in histopathological diagnosis was noted. A histopathological diagnosis was unavailable in 12% of tumors after pathologist review. Similar findings were reported in a recent review of neuro-oncology articles from East Africa: 14% of tumors were without a general histologic description and 32% of tumors were reported as unknown or no specified diagnosis (11). Also a fraction of patients (10%) who underwent surgery did not receive a histopathology report despite having their tissue samples sent to the pathologist. Some study sites described delays in receiving histopathology reports taking an average of 4-5 weeks to receive the report post-surgery. The proportion of cases without a histopathology diagnosis indicates areas for improvement in tissue acquisition, processing, analysis, and data storage.

Many patients did not receive standard, comprehensive neuro-oncology care due to poor access or inadequate resources. About a third of cases did not receive surgery and a majority of patients (74%-85%) for whom adjuvant therapy was indicated based on their histopathology diagnosis did not receive adjuvant therapy. In the few patients who are able to receive adjuvant care, dyscoordination between neurosurgery and oncology teams during or following neurosurgical treatment often leads to a delayed start of adjuvant chemotherapy and/or radiotherapy or patients altogether missing the optimal window to receive adjuvant therapy (11, 17). The limited surgical infrastructure and neurocritical care coupled with cost of care contribute to prolonged wait times observed at these study sites relative to well-equipped treatment centers in other countries (28, 29). A meta-analysis evaluating differences in postoperative LOS after brain tumor surgery in HICs and LMICs showed a postoperative LOS in LMICs (10.1 days) similar to what we report in our study (10.6 days) which is longer than the postoperative LOS in HICs (5.1 days) (30). Factors accounting for longer postoperative LOS in LMICs include poorly treated comorbidities, postoperative complications, and lack of adoption of contemporary and/or minimally invasive neurosurgical approaches and techniques due to limited infrastructure (31). While this study does not compare clinical practice in HICs and LMICs, we understand historically and anecdotally there is dramatic inequity in the delivery and reception of care for pediatric CNS tumor patients between these income categories (10, 11). To help address the existing cancer health disparities between HICs and LMICs, the International Society of Pediatric Oncology (SIOP) is currently leading efforts to develop clinical guidelines for different pediatric cancers including CNS tumors based on the resources and facilities available in LMICs (32–34).

Considering the otherwise vast catchment area, the relatively modest burden of cases reported over the 9-month study period also highlights the underdiagnosis and underreporting of neuro-oncologic cases in developing countries (9). Much of this is attributed to limited access to proper neurologic assessments and neuroimaging needed to make a definitive diagnosis (35). As a subcontinental collaborative, we are conducting a follow-up study to better elucidate these and other underlying disparities, and to propose site-derived solutions.

There are several limitations to this study. This is a cross sectional survey and therefore indicates only the neurosurgical practice pattern during the specific 9-month period. As the goal of this study was to determine an epidemiologic profile of tumors managed at these national referral sites, patient-specific details were not captured. These figures represent hospital-level data; population incidence and prevalence cannot be inferred by this methodology. Also, the design of our study did not seek to obtain long-term follow-up data. We used a snowball sampling approach to identify research collaborators as there is currently no existing catalog of pediatric CNS tumor treatment centers in SSA and details on the types of cancers managed at those centers. Thus, while the 7 sites themselves represent large and diverse catchment areas, these results cannot be generalized to all regions in SSA due to selection bias.

## Conclusion

The hospital-level burden, histopathology, and location of pediatric brain and spinal tumors managed at seven referral sites in Sub-Saharan Africa is described. Among patients requiring surgical care, two-thirds received a tumor-directed surgery; less than a third received indicated adjuvant chemotherapy or radiation. Significant healthcare investments are needed to build diagnostic infrastructure, enhance surgical capabilities, and offer adjuvant therapy for children in SSA diagnosed with a CNS neoplasm.

## Data availability statement

The raw data supporting the conclusions of this article will be made available by the authors, without undue reservation.

## Ethics statement

Institutional review board (IRB) approval was obtained from all 7 SSA sites, as well as the North American coordinating site, Vanderbilt University Medical Center. The seven SSA study sites included: Aminu Kano Teaching Hospital (AKTH), Kano, Nigeria; Komfo Anokye Teaching Hospital (KATH), Kumasi, Ghana; Nelson Mandela Children’s Hospital (NMCH), Johannesburg, South Africa; Tenwek Mission Hospital(TMH), Bomet, Kenya; Zewditu Memorial Hospital (ZMH), Addis Ababa, Ethiopia; University Teaching Hospital (UTH), Lusaka, Zambia; and Muhimbili Orthopedic Institute (MOI), Dar es Salaam, Tanzania. Written informed consent from the participants was not required to participate in this study in accordance with the national legislation and the institutional requirements.

## Author contributions

JH-C: Conceptualization, Data curation, Formal Analysis, Methodology, Project administration, Resources, Software, Validation, Writing – original draft, Writing – review & editing. SC: Conceptualization, Data curation, Formal Analysis, Funding acquisition, Methodology, Resources, Software, Validation, Writing – original draft, Writing – review & editing. JL: Conceptualization, Data curation, Methodology, Writing – review & editing. AB: Conceptualization, Data curation, Methodology, Writing – review & editing. YA: Conceptualization, Data curation, Methodology, Writing – review & editing. HS: Conceptualization, Data curation, Methodology, Writing – review & editing. WC: Conceptualization, Data curation, Methodology, Writing – review & editing. KS: Conceptualization, Data curation, Methodology, Writing – review & editing. MA: Conceptualization, Data curation, Methodology, Writing – review & editing. FN-B: Conceptualization, Data curation, Methodology, Writing – review & editing. MD: Conceptualization, Data curation, Formal Analysis, Funding acquisition, Methodology, Resources, Software, Supervision, Writing – review & editing.
