# Supplementary material for: Pediatric neurosurgical-oncology scope and management paradigms in Sub-Saharan Africa: a collaboration among 7 referral hospitals on the subcontinent
Source: Front Oncol. 2023 Nov 1;13:1257099. doi: 10.3389/fonc.2023.1257099 (PMC10646489; doi:10.3389/fonc.2023.1257099)
Supplement: Supplementary file 1 [file DataSheet_1.pdf]

Neurooncology Epidemiology Survey

All the survey questions refer to the pediatric population patients aged less than 18 years.

Hospital and Surgeon related information

Please select the location of hospital

- ☐ South Africa: Johannesburg
- ☐ Ethiopia: Addis Ababa
- ☐ Tanzania: Dar es Salaam
- ☐ Kenya: Bomet
- ☐ Zambia: Lusaka
- ☐ Nigeria: Kano
- ☐ Ghana: Kumasi
- ☐ Uganda: Kampala

Name of hospital

____

Please provide your role.

- ◯ Care Partner (non-physician)
- ◯ Neurosurgeon
- ◯ Other

Have you received formal pediatric neurosurgery training (e.g. fellowship), above and beyond the required training to become a general neurosurgeon?

- ◯ Yes
- ◯ No

Total neurosurgeons at your hospital with pediatric neurosurgery practice, including yourself?

- ◯ 1
- ◯ 2
- ◯ 3
- ◯ 4
- ◯ 5
- ◯ 6
- ◯ 7
- ◯ 8
- ◯ 9
- ◯ 10

Note: Neuro-Oncology refers to tumors of the brain and spine

What percentage of your center's out-patient/clinic volume is represented by pediatric neuro-oncology? (%)

(Place a mark on the scale above)

What percentage of your center's surgical/operative volume is represented by pediatric neuro-oncology? (%)

(Place a mark on the scale above)

Does your center have the following diagnostic modalities?

- ◯ CT scan only
- ◯ MRI only
- ◯ Both
- ◯ None

Does your center have one of the following radiologist to read the images?

- ◯ Neuroradiologist
- ◯ General radiologist
- ◯ None

Among patients who undergo surgery for tumor resection, what percentage undergo preoperative CT scan? (%)

(Place a mark on the scale above)

Among patients who undergo surgery for tumor resection, what percentage undergo preoperative MRI scan? (%)

(Place a mark on the scale above)

Among patients who undergo surgery for tumor resection, what percentage undergo immediate or early post-operative CT scan? (%)

(Place a mark on the scale above)

Among patients who undergo surgery for tumor resection, what percentage undergo immediate/early postoperative MRI scan? (%)

(Place a mark on the scale above)

Tumor-related information
(Reminder: All the survey questions refer to the pediatric population patients aged less than 18 years).

Number of pediatric patients with a brain or spinal tumor diagnosis presenting to the clinic or emergency department in the last 3-months? (here: pediatric = age less than 18 years). Please provide a number. ______________________

How many pediatric brain or spinal tumors were operated on in the last 3-months? Please provide a number. ______________________

Total number of patients who underwent biopsy in the last 3 months. ______________________

Total number of patients who underwent resection (including subtotal and total) in the last 3 months. ______________________

In the last 3-months, for patients seen in ED or clinic with a new diagnosis of brain or spinal tumor, what was the mean (average) time to surgery? (excluding those patients who did not undergo surgery). (days) ______________________

In the last 3-months, for patients admitted to the hospital/ward with a new diagnosis of brain or spinal tumor, what was the mean (average) time to surgery? (excluding those patients who did not undergo surgery). (days)	______________________
In the last 3-months, among patients who did not undergo surgery for a brain or spinal tumor, what percentage (%) should have received surgery but did not undergo surgery because of inadequate resources (OR time, ICU beds, surgeon(s), equipment, blood products, financial compensation, etc).	*(Place a mark on the scale above)*
Total number of patients with brain or spine tumor referred to another center in the past 3-months?	______________________
Please select the predominant radiographic location of tumors at your center (including yourself and your partners) evaluated in the last 3-months? Select all that applies and provide number of tumors seen in each location. If a tumor occupies more than one designated location, select the predominant location.	☐ Frontal<br>☐ Parietal<br>☐ Temporal<br>☐ Occipital<br>☐ Thalamic<br>☐ Insular<br>☐ Sellar/Suprasellar<br>☐ Optic pathway<br>☐ Tectal<br>☐ Brainstem<br>☐ Posterior fossa<br>☐ Ventricular<br>☐ Spinal<br>☐ Pineal region<br>☐ Other
Frontal (#)	______________________
Parietal (#)	______________________
Temporal (#)	______________________
Occipital (#)	______________________
Thalamic (#)	______________________
Insular (#)	______________________
Sellar/Suprasellar (#)	______________________
Optic Pathway (#)	______________________

Tectal (#) ______________________

Brainstem (#) ______________________

Posterior fossa (#) ______________________

Ventricular (#) ______________________

Spinal (#) ______________________

Pineal region (#) ______________________

Other (provide location) ______________________

Other (#) ______________________

Surgery-related questions
(Reminder: All the survey questions refer to the pediatric population patients aged less than 18 years).

Please select the radiographic location of tumors at your unit (including yourself and your partners) operated on in the last 3 months? Select all that applies and provide number of tumors operated on in each location

- ☐ Frontal
- ☐ Parietal
- ☐ Temporal
- ☐ Occipital
- ☐ Thalamic
- ☐ Insular
- ☐ Sellar/Suprasellar
- ☐ Optic pathway
- ☐ Tectal
- ☐ Brainstem
- ☐ Posterior fossa
- ☐ Ventricular
- ☐ Spinal
- ☐ Pineal region
- ☐ Other

Frontal (#) ______________________

Parietal (#) ______________________

Temporal (#) ______________________

Occipital (#) ______________________

Thalamic (#)	______________________
Insular (#)	______________________
Sellar/Suprasellar (#)	______________________
Optic Pathway (#)	______________________
Tectal (#)	______________________
Brainstem (#)	______________________
Posterior fossa (#)	______________________
Ventricular (#)	______________________
Spinal (#)	______________________
Pineal region (#)	______________________
Other (provide location)	______________________
Other (#)	______________________
Which of the following pathologic capabilities does your center have? (select all that apply)	☐ Basic histopathology (H&E stains).<br>☐ Advanced histopathology stains.<br>☐ Neuropathology expertise.<br>☐ Molecular subtyping.<br>☐ Intraoperative frozen section (quick section).

Please select the histopathologic types of tumors operated on in the last 3-months? Select all that applies and provide number of tumors operated on in each location.	☐ Low grade glioma<br>☐ High-grade glioma<br>☐ DNET (dysembryoplastic neuroepithelial tumor)<br>☐ Oligodendroglioma<br>☐ Ependymoma<br>☐ Subependymoma<br>☐ Medulloblastoma<br>☐ Atypical teratoid rhabdoid tumor (ATRT)<br>☐ Craniopharyngioma<br>☐ Meningioma<br>☐ Schwannoma<br>☐ Adenoma<br>☐ Metastasis<br>☐ Epidermoid<br>☐ Dermoid<br>☐ Germ cell tumors<br>☐ Neoplasm: histopathologic diagnosis unknown<br>☐ Other
Low grade glioma (#)	______________________
High grade glioma (#)	______________________
DNET (#)	______________________
Oligodendroglioma (#)	______________________
Ependymoma (#)	______________________
Subependymoma (#)	______________________
Medulloblastoma (#)	______________________
ATRT (#)	______________________
Craniopharyngioma (#)	______________________
Meningioma (#)	______________________
Schwannoma (#)	______________________
Adenoma (#)	______________________
Metastasis (#)	______________________

Epidermoid (#) ______________________

Dermoid (#) ______________________

Germ cell tumor (#) ______________________

Neoplasm: histopathologic diagnosis unknown (#) ______________________

Other ______________________

Total number of patients with MINOR postoperative complications (for example: post-op infection not requiring surgery, wound dehiscence/complication, transient/mild neurologic deficit) associated with pediatric brain and spine tumor surgeries in the past 3-months. ______________________

Total number of patients with MAJOR postoperative complications (for example: hemiparesis, hemiplegia, permanent aphasia, post-op meningitis, return to OR for surgical complication) associated with pediatric brain tumor surgeries in the past 3-months. ______________________

Of those patients who underwent brain tumor resection in the last 3-months, how many required a CSF diversion (either CSF shunt or ETV)? ______________________

In the last 3-months, how many patients with a diagnosis of brain tumor and hydrocephalus underwent CSF diversion (either CSF shunt or ETV) alone, without surgical resection of the tumor? ______________________

Total number of deaths associated with brain or spinal tumor patients in the last 3-months. ______________________

Total number of deaths associate with brain or spinal tumor patients who underwent surgery in the last 3-months. ______________________

Do you or your institution host a "Tumor Board" (also known as Tumor Conference or Neuro-Oncology Conference) wherein a multi-disciplinary team of physicians (neurosurgeons, oncologists, pathologists, radiologists, and/or radiation oncologists) meet on a regular basis to discuss brain & spinal tumor patients and their diagnosis, surgery, and any plans for adjuvant therapy (eg. chemotherapy, radiation, etc) or observation?

- ◯ Yes
- ◯ No

How often does the multi-disciplinary team meet? ______________________

Would you or your institution be interested in forming a Tumor Board, with or without virtual assistance from outside institutions?

◯ Yes
◯ No

In the last 3-months, what percentage (%) of patients whose diagnosis calls for adjuvant CHEMOTHERAPY actually received such adjuvant care?

(Place a mark on the scale above)

In the last 3-months, what percentage (%) of patients whose diagnosis calls for adjuvant RADIOTHERAPY actually received such adjuvant care?

(Place a mark on the scale above)

Mean (average) length of preoperative hospital stay for patients who underwent tumor resection in the last 3-months. (days)

____

Mean (average) length of post-operative hospital stay for patients who underwent tumor resection in the last 3-months. (days)

____

Mean (average) length of ICU stay for patients who underwent tumor resection in the last 3-months. (days)

____

What percentage (%) of patients who underwent brain or spinal tumor surgery, were discharged from the hospital, and followed up with an early (2-6 weeks) postoperative clinic appointment?

(Place a mark on the scale above)

What percentage (%) of patients who underwent brain or spinal tumor surgery, were discharged from the hospital, and followed up with a long-term (> 3months) postoperative clinic appointment? (Use the previous 3-month's information to answer this question about long-term follow-up)

(Place a mark on the scale above)

Wait-list related questions

(Reminder: All the survey questions refer to the pediatric population patients aged less than 18 years).

Does your center have a waiting list for pediatric neuro oncology?

◯ Yes
◯ No

In the last 3-months, how many children with brain or spinal tumor died or had major complication while waiting for surgery? (please include those that are in or outside of the neurosurgical wards).

____

In the last 3-months, provide the three most common reasons for delay of definitive treatment for brain or spinal tumors?

____

Miscellaneous Questions
(Reminder: All the survey questions refer to the pediatric population patients aged less than 18 years).

Is HIV serology testing routinely done in your practice?
- ◯ Yes
- ◯ No

If yes, does how does it affect your management?

____

In the last 3-months, how has COVID impacted your center's brain or spine tumor surgery volume?
- ◯ No major reduction in volume
- ◯ Reduced by about 25%
- ◯ Reduced by about 50%
- ◯ Reduced by about 75%
- ◯ Reduced by about > 75%

In the last 3-months, among patients with a brain or spinal tumor, on average how many complications or deaths related to COVID occurred at your center?

____

Is there anything you would like to explain or add regarding your answers to the questions in this survey?

____

With the advent of newer technology that allows virtual reality and virtual presence for surgical education and training, would you or your institution be interested in exploring this concept? This technology uses low bandwidth internet, cameras, and audiovisual communications to allow surgeons across different institutions and countries to interact with each other in the operating theatre in real-time.
- ◯ Yes
- ◯ No

What would you find this technology to be most beneficial for?
- ☐ HIC-LMIC surgical education and training?
- ☐ LMIC-LMIC surgical education and training?
- ☐ Some other form of training that could be useful to your institutions

Please specify

____
